# Evaluation of plasma prealbumin as a novel inflammatory biomarker in dogs: a pilot study

**DOI:** 10.3389/fvets.2023.1142535

**Published:** 2023-05-24

**Authors:** Sin-Wook Park, Keon Kim, Ock-Kyu Kim, Woong-Bin Ro, Chang-Min Lee

**Affiliations:** ^1^Department of Veterinary Internal Medicine, College of Veterinary Medicine and BK21 FOUR Program for Creative Veterinary Science Research Center, Chonnam National University, Gwangju, Republic of Korea; ^2^Cat Vet Animal Hospital, Seongnam-si, Gyeonggi-do, Republic of Korea

**Keywords:** canine, biomarkers, inflammation, negative acute phase protein, transthyretin

## Abstract

**Introduction:**

Prealbumin (PAB) is a plasma protein synthesized in the hepatic parenchymal cells. PAB has a short half-life (~2 days), and its concentration is affected by changes in transcapillary escape. Measurement of PAB is widely used in hospitalized patients in human medicine due to its decreasing concentration in states of inflammation and malnutrition. However, only a few studies are available in dogs. The aim of this study is to determine whether the plasma PAB concentration decreases in dogs with inflammation and to evaluate the relationship between the plasma PAB concentration and inflammation-related parameters in dogs.

**Methods:**

A total of 94 dogs were divided into healthy (*n* = 33) and diseased (*n* = 61) groups. These were further divided into group A (*n* = 24) and group B (*n* = 37) according to plasma C-reactive protein (CRP) levels. Group A included dogs with a plasma CRP < 10 mg/L, and group B consisted of dogs with a plasma CRP ≥ 10 mg/L. Patient signalment, history, physical examination findings, hematologic and biochemical parameters, various inflammatory markers, and plasma PAB levels were investigated and compared between groups.

**Results:**

The plasma PAB concentration was found to be lower in group B than in the other groups (*p* < 0.001), but no statistical difference was found when comparing the control group and group A (*p* > 0.05). A plasma PAB < 6.3 mg/dL predicted an increased CRP level (10 mg/L or greater) with a sensitivity of 89.5% and a specificity of 86.5%. Receiver operating characteristic curve analysis revealed that the area under the curve for PAB was higher than that for the white blood cell count, neutrophil count, albumin level, lactate level, neutrophil-to-lymphocyte ratio, and neutrophil percentage-to-albumin ratio. In addition, the PAB concentration was significantly negatively correlated with the CRP concentration (*r* = −0.670, *p* < 0.001).

**Conclusion:**

In conclusion, this is the first study to demonstrate the clinical usefulness of the plasma PAB concentration as an inflammatory marker in dogs. These findings suggest that measuring the plasma PAB concentration along with the CRP concentration may be more useful for evaluating inflammation than measuring CRP alone in canine patients.

## Introduction

Prealbumin (PAB), also known as transthyretin, is a protein that contains 127 amino acid residues (~13,800 Da) and is mainly synthesized by hepatocytes. It is called PAB because it migrates faster than albumin on serum electrophoresis (1, 2). PAB is an important transport protein in blood, carrying thyroid hormones and indirectly transporting vitamin A (1).

PAB is known to be a more sensitive indicator of malnutrition than albumin because it has a shorter half-life of approximately 2.5 days and faster synthesis in hepatocytes (3). With these characteristics, PAB is used to evaluate malnutrition in hospitalized patients (4). In addition, the plasma PAB concentration is significantly affected by inflammatory conditions such as trauma, tissue injury, and stress as a negative acute phase protein (APP) in humans (5–8).

When inflammation occurs due to trauma, infection, tissue damage, or tumors, pro-inflammatory cytokines increase, and other inflammatory pathways are activated, causing various effects on the body (9, 10). Rapid detection of inflammation is important for evaluating the treatment and prognosis of a patient; thus, various indicators related to inflammation have been studied. There are some parameters that could be useful for evaluating inflammation such as C-reactive protein (CRP) level, albumin concentration, white blood cell (WBC) count, neutrophil (NEU) count, lactate level, neutrophil-to-lymphocyte ratio (NLR), platelet-to-lymphocyte ratio (PLR), monocyte-to-lymphocyte ratio (MLR), and neutrophil percentage-to-lymphocyte ratio (NPAR) in dogs (9, 11–13). Among them, CRP is a positive APP that increases by 100–1,000 times within 24–48 h in an inflammatory state, decreases rapidly when inflammation is resolved, and is one of the most widely used indicators for confirming inflammation in dogs due to its high sensitivity and specificity (9, 14).

In human medicine, the PAB concentration reflects inflammatory conditions just as well as CRP (15). In addition, PAB is used as prognostic indicator that correlates well with the clinical prognosis of the disease condition as well as the nutritional status of the patient (16). However, no study has evaluated PAB as an inflammatory marker in dogs.

The plasma PAB concentration has been suggested as a useful biomarker for monitoring inflammation in dogs. The objective of this study was to determine whether the plasma PAB concentration is decreased in dogs with inflammation and to evaluate the relationship between the PAB concentration and inflammation-related parameters in dogs.

## Materials and methods

### Animals

A total of 94 client-owned dogs that presented to Chonnam National University Veterinary Teaching Hospital and Gwangju Sky Animal Medical Center between January 2022 and August 2022 were investigated.

The control group included 33 healthy dogs presented for routine examinations or elective procedures (e.g., castrations). Dogs were enrolled if they were older than 6 months old, clinically healthy, and without clinical evidence of disease based on physical examination, complete blood count (CBC), and plasma biochemical test results and had no history of anorexia.

Sixty-one dogs with abnormalities in their patient history, physical examination, CBC, and plasma biochemical test results were enrolled. Based on the plasma CRP concentration, diseased dogs were divided into group A and group B. Group A consisted of dogs with a plasma CRP < 10 mg/L. Group B consisted of dogs with a plasma CRP ≥ 10 mg/L.

The exclusion criteria included a body condition score ≤ 2/9 and attending clinician determination that the dog was unstable for participation (17). Based on hematology and diagnostic imaging examinations (X-ray, ultrasonography, or computed tomography), dogs with hepatic diseases such as acute hepatitis, chronic hepatitis, portosystemic shunts, or cirrhosis were excluded. In addition, dogs with diseases associated with protein loss (protein-losing enteropathy and protein-losing nephropathy) determined based on hematology, urinalysis, ultrasonographic examination, and biopsy were excluded.

### Blood sampling and analysis

Blood was taken from the cephalic or jugular vein from fasted dogs on the first presentation. Blood samples for performing a CBC were collected into tubes containing the anticoagulant ethylenediaminetetraacetic acid. Blood samples for determining biochemical parameters and the CRP profile were collected in heparin tubes. Blood samples collected in heparin tubes were centrifuged for 5 min at 4000 × g. A CBC, biochemical analyses, and CRP levels were measured within 30 min after collection of the blood samples. A plasma chemistry analyzer (Catalyst Dx Chemistry Analyzer, IDEXX Veterinary Diagnostics, Co., United States) was used to quantify serological parameters. CBC were measured using a hematology analyzer (Procyte Dx Analyzer, IDEXX Veterinary Diagnostics, Co., United States). Plasma samples were stored for a maximum of 6 months at −20°C for analysis.

### Measurement of plasma PAB

A PAB assay was performed by COBAS C702 automated analyzer (Integra; Roche, Basel, Switzerland) using PAB reagent (PREA: ACN 8710; Basel, Switzerland). According to the manufacturer, this PAB assay has a detection limit of 3 mg/dL at low concentrations.

### Measurement of plasma CRP and lactate

A CRP assay was performed using an automatic chemistry colorimetric analyzer (Dotto 2000 Auto Chemistry Analyzer, MTD Diagnostics Co., Italy) using canine CRP reagent and lactate reagent (LC Diagnostics, Co., South Korea). Evidence of inflammation was defined as a plasma CRP ≥ 10 mg/L according to the manufacturer.

### Statistical analysis

All statistic data were analyzed using commercial software (IBM SPSS Statistics, version 25, IBM Co., United States). Descriptive statistics are reported as the mean ± standard deviation (SD) and median (interquartile range, IQR). To assess for differences between groups, the plasma level of PAB, CRP, albumin, lactate, WBC count, NEU count, NLR, MLR, PLR, and NPAR were analyzed with Welch’s ANOVA and the Dunnett T3 *post hoc* test. In addition, the differences between these parameters among groups were analyzed with the Kruskal-Wallis test. Multiple Mann–Whitney *U* tests with Bonferroni correction were performed as *post hoc* tests after which *p* < 0.05 was defined as representing statistical significance.

The correlations between the plasma level of PAB, CRP and other inflammation-related parameters were evaluated using Spearman’s rank correlation. Receiver operator characteristic (ROC) procedure and its area under the curve (AUC) were used to determine a cutoff value for inflammation-related parameters (PAB, WBC, NEU, albumin, lactate, NLR, MLR, and NPAR) for differentiation of inflammation (plasma CRP ≥ 10 mg/L) in all dogs. Statistical significance was considered as *p* < 0.05 for all tests applied.

## Results

Ninety-four client-owned dogs of 13 different breeds were included in the study. Ten dogs were mixed breeds. The most common breeds were Maltese (*n* = 27), Pomeranian (*n* = 16), Miniature Poodle (*n* = 10), Shih Tzu (*n* = 9), Bichon Frise (*n* = 5), Yorkshire Terrier (*n* = 4) and Beagle (*n* = 4). Other breeds were represented with less than three dogs. The mean age at presentation was 7.7 ± 3.6 years (range, 11 months to 16 years). Forty dogs were male (42.5%), and 34 of them were neutered; 43 dogs were female (57.5%), and 35 of them were spayed. The mean body weight of all dogs was 4.65 ± 2.29.

Thirty-three healthy dogs were included in the control group. Sixty-one dogs with diseases were divided into group A (*n* = 24) and group B (*n* = 37). Further diagnostic tests were performed to confirm the diagnoses and to detect the severity of disease (e.g., diagnostic imaging tests, cytologic or histologic examination of tumors and purulent material collected from the uterus).

In group A, the dogs were diagnosed with mitral valve insufficiency (*n* = 6), mammary gland tumors (WHO stage I, *n* = 4), intravertebral disk protrusion (*n* = 4), atopic dermatitis (*n* = 3), and miscellaneous diseases (*n* = 7). In group B, the dogs were diagnosed with acute pancreatitis (*n* = 12), pyometra (*n* = 11), malignant tumors with metastasis (*n* = 5), and miscellaneous diseases (*n* = 9). Malignant tumors with metastasis included hemangiosarcoma (*n* = 2), small intestinal adenocarcinoma (*n* = 1), mast cell tumor (*n* = 1), and renal carcinoma (*n* = 1). Miscellaneous diseases included those that did not belong to any of the abovementioned disease categories.

**Figure 1:**
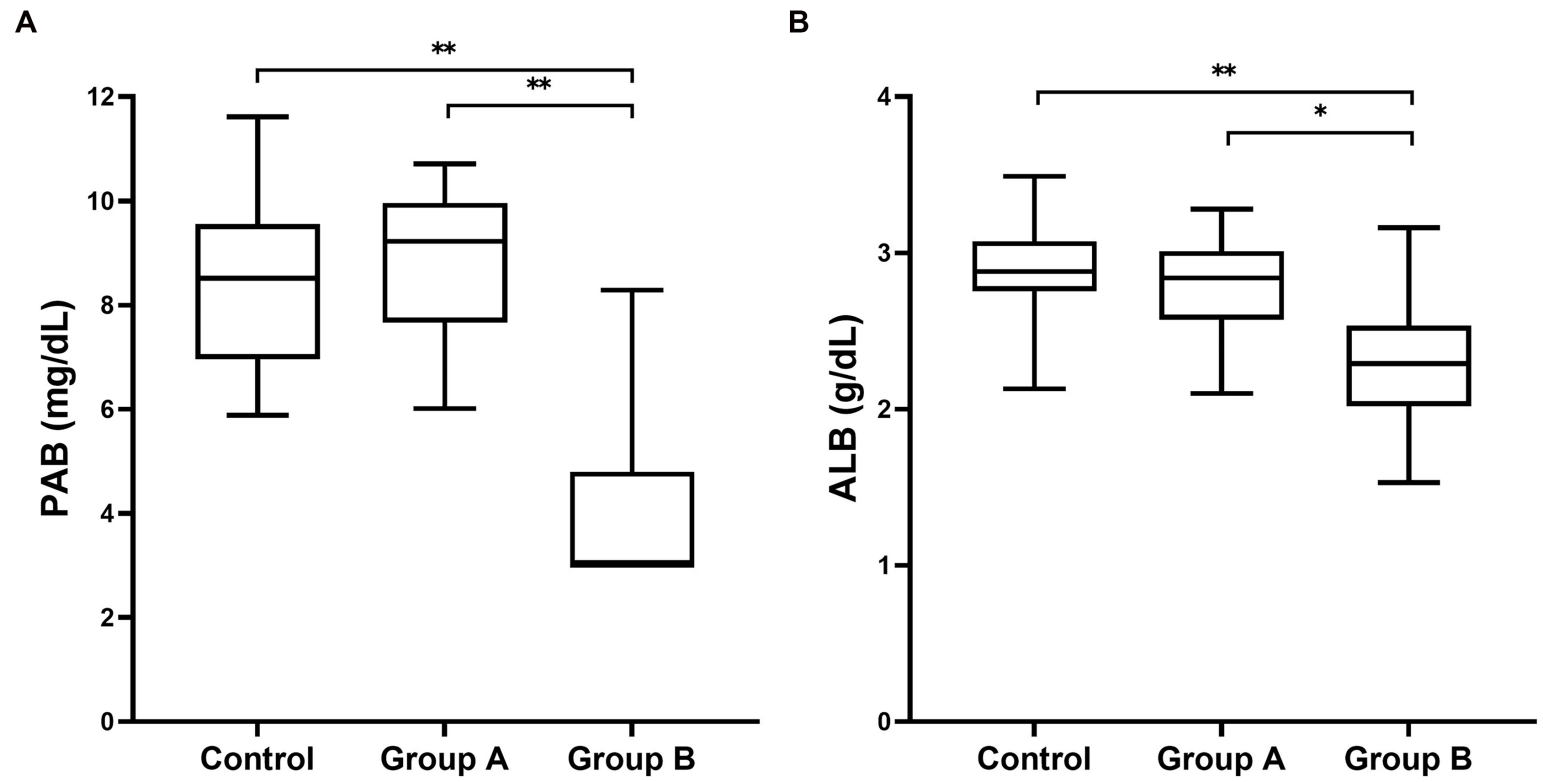
Box and whisker plot of plasma PAB **(A)** and albumin **(B)** concentrations in dogs included in this study. Boxes extend from the 25th percentile to the 75th percentile. The horizontal bar in the box represents the median. The whiskers represent the range. PAB concentration less than 3 mg/dL represents the lowest measurable analyte level (3 mg/dL) that can be distinguished from zero. ^*^ *p* < 0.01 and ^**^ *p* < 0.001 indicate a statistically significant difference.

Evaluation of inflammatory biomarkers and PAB was performed in all dogs. Their values on admission for all groups are presented in Table 1. A plasma PAB concentration < 3 mg/dL is indicated by 3 mg/dL. There were dogs with a plasma PAB concentration of 3 mg/dL in group B (22/37), while there were no dogs with a plasma PAB concentration of 3 mg/dL in the other groups. The plasma PAB and albumin concentrations showed no significant differences between the control group and group A (*p* > 0.05). However, the PAB and albumin concentrations were significantly decreased in group B compared with both the control group and group A (*p* < 0.05). In addition, a larger decrease in the median plasma PAB concentration than in the albumin concentration was found (Figure 1).

**Table 1:** Evaluation of inflammatory biomarkers and PAB in the control group and groups A and B.

Variable (Reference range)	Total dogs (*N* = 94) Mean ± SD or Median (IQR)				Adjusted *p* value for *post hoc* significance if indicated		
	Control (*N* = 33)	Group A (*N* = 24)	Group B (*N* = 37)	*p* value	Control versus Group A	Control versus Group B	Group A versus Group B
PAB (mg/dL)	8.51 (7.16–9.55)	9.22 (7.99–9.55)	3 (3–4.52)	<0.001^***^	0.110	<0.001^***^	<0.001^***^
CRP (0–10 mg/L)	0.3 (0.2–0.6)	1.9 (0.6–5.9)	63 (35.8–106.8)	<0.001^***^	<0.001^***^	<0.001^***^	<0.001^***^
ALB (2.2–3.9 g/dL)	3.3 (3.1–3.6)	3.1 (2.8–3.4)	2.7 (2.4–3)	<0.001^***^	0.139	<0.001^***^	0.004^**^
WBC (5.05–16.76 K/uL)	8.34 (6.5–10.17)	8.71 (7.5–10.05)	18.5 (10.76–25.9)	<0.001^***^	0.465	<0.001^***^	<0.001^***^
NEU (2.95–11.64 K/uL)	5.2 (4.4–5.7)	6.1 (5.3–7.5)	14 (8.6–21.4)	<0.001^***^	0.017	<0.001^***^	<0.001^***^
LAC (0–25 mg/dL)	16.8 (10.3–27.8)	29 (19–37.9)	44.8 (35.3–65.4)	<0.001^***^	0.021^*^	<0.001^***^	<0.001^***^
NLR	2.28 (1.84–3.06)	3.64 (2.15–5.06)	4.94 (2.62–8.21)	0.003^**^	0.024^*^	0.001^**^	0.179
MLR	0.23 (0.2–0.31)	0.3 (0.21–0.44)	0.39 (0.21–0.62)	0.044^*^	0.143	0.014^*^	0.367
PLR	149.7 (99.5–205.2)	276.6 (215.5–77.6)	140 (72.4–293.9)	0.006^**^	0.001^**^	0.978	0.014^*^
NPAR	18.8 (17.2–21.1)	22.3 (19.3–26.7)	27.2 (22.1–36.0)	<0.001^***^	0.009^**^	<0.001^***^	0.036^*^

PAB, prealbumin; CRP, C-reactive protein; WBC, white blood cell; NEU, neutrophil; NLR, neutrophil-to-lymphocyte ratio; MLR, monocyte-to-lymphocyte ratio; PLR, platelet-to-lymphocyte ratio; NPAR, neutrophil percentage-to-albumin ratio. Values marked with ^*^ *p* < 0.05, ^**^p < 0.01 and ^***^ *p* < 0.001 indicates a statistically significant difference. Parametric data are reported as median and interquartile ranges. *p* values were analyzed with Welch’s ANOVA test.

The CRP level, WBC count, NEU count, lactate level, and NPAR were significantly higher in group B than in the other groups (*p* < 0.05). The CRP level, lactate level, NLR, and NPAR were lower in the control group than in groups A and B (*p* < 0.05). Among them, the CRP level, lactate level, and NPAR showed significant differences among the three groups (*p* < 0.05).

The correlations between the plasma PAB level and inflammation-related parameters are reported in Table 2. In addition, the correlations between the plasma CRP level and inflammation-related parameters are shown in Table 3. Among inflammation-related parameters, the CRP level showed the most significant correlation with the PAB concentration (*r* = −0.670, *p* < 0.001).

**Table 2:** Correlation between PAB and inflammation-related parameters in all dogs.

Inflammation-related parameters	PAB	
	*r*	*p*
CRP	−0.670	<0.001
Albumin	0.386	<0.001
WBC	−0.520	<0.001
NEU	−0.541	<0.001
Lactate	−0.429	<0.001
NLR	−0.272	0.014
MLR	−0.191	0.087
PLR	0.125	0.254
NPAR	−0.282	0.012

PAB, Prealbumin; CRP, C-reactive protein; WBC, White blood cell; NLR, Neutrophil-to-lymphocyte ratio; NEU, Neutrophil; MLR, Monocyte-to-lymphocyte ratio; PLR, Platelet-to-lymphocyte ratio; NPAR, Neutrophil percentage-to-albumin ratio.

**Table 3:** Correlation between CRP and inflammation-related parameters in all dogs.

Inflammation-related parameters	CRP	
	*r*	*p*
PAB	−0.670	<0.001
Albumin	−0.428	<0.001
WBC	0.625	<0.001
NEU	0.542	<0.001
Lactate	0.347	0.001
NLR	0.313	<0.001
MLR	0.271	0.015
PLR	−0.054	0.624
NPAR	0.204	0.073

CRP, C-reactive protein; PAB, Prealbumin; WBC, White blood cell; NEU, Neutrophil; NLR, Neutrophil-to-lymphocyte ratio; MLR, Monocyte-to-lymphocyte ratio; PLR, Platelet-to-lymphocyte ratio; NPAR, Neutrophil percentage-to-albumin ratio.

There were statistically significant correlations between the plasma PAB concentration and the CRP level, albumin concentration, WBC count, NEU count, and lactate level (*p* < 0.05). The plasma PAB concentration was positively correlated with the albumin concentration and PLR. The CRP level, WBC count, NEU count, lactate level, NLR, MLR, and NPAR showed negative correlations with the plasma PAB concentration. In addition, there were statistically significant correlations between the plasma CRP level and the PAB concentration, albumin concentration, WBC count, NEU count, lactate level, NLR, and MLR (*p* < 0.05). The plasma CRP level was positively correlated with the WBC count, NEU count, lactate level, NLR, MLR, and NPAR. Finally, the plasma CRP level was negatively correlated with the plasma PAB concentration, albumin concentration, and PLR.

**Figure 2:**
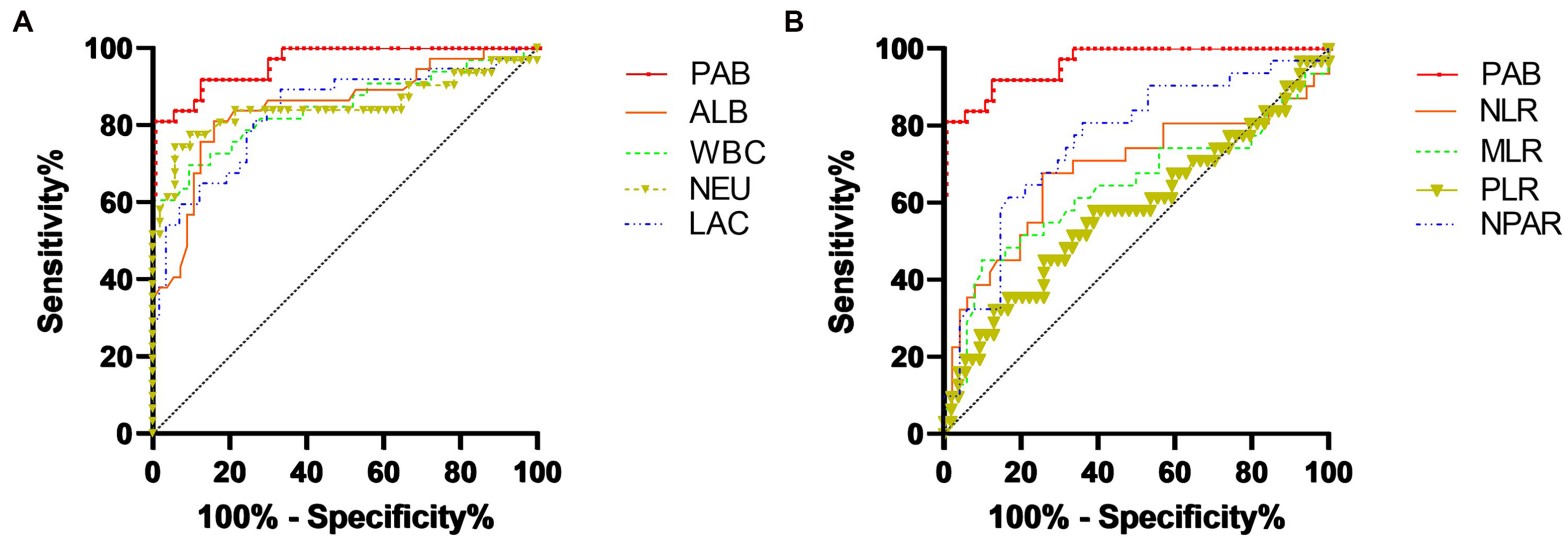
ROC curve analyses to determine the thresholds for the PAB concentration, albumin concentration, WBC count, NEU count, and lactate level for predicting an increased CRP level (≥10 mg/dL) **(A)**. ROC curve analysis determined the thresholds for the PAB concentration, NLR, MLR, PLR, and NPAR for predicting an increased CRP level (≥10 mg/dL) **(B)**. A PAB concentration less than 6.3 mg/dL predicted an increased CRP level (plasma CRP ≥ 10 mg/L) with a sensitivity of 89.5% and a specificity of 86.5%.

The results of the ROC curve analyses and AUC values to predict inflammation (CRP ≥ 10 mg/L) in all dogs are summarized in Table 4. The PAB concentration showed the highest area AUC (0.95) compared with other inflammation-related parameters in this study (Figure 2). A plasma PAB concentration less than 6.3 mg/dL predicted inflammation (plasma CRP ≥ 10 mg/L) with a sensitivity of 89.5% and a specificity of 86.5%.

**Table 4:** ROC curve analysis for determination of the cutoff value for PAB, WBC, NEU, ALB, LAC, NLR, PLR, MLR, and NPAR as predictor of inflammation status (CRP concentration 10 mg/L or greater) in all dogs.

Variable	AUC	95% CI	*p* value	Cutoff value	Sensitivity (%)	Specificity (%)
PAB	0.95	0.9–1	<0.001^***^	6.3 mg/L	89.5	86.5
ALB	0.79	0.69–0.9	<0.001^***^	3 g/dL	71.4	75.7
WBC	0.85	0.76–0.95	<0.001^***^	10.2 K/uL	77.4	76.6
NEU	0.84	0.74–0.95	<0.001^***^	7.2 K/uL	80.6	83
LAC	0.87	0.78–0.95	<0.001^***^	33 mg/dL	80.6	80.9
NLR	0.68	0.55–0.81	0.005^**^	3.9	67.7	68.1
MLR	0.64	0.5–0.77	0.007^*^	0.32	61.3	66
PLR	0.59	0.56–0.73	0.178	200	50	50
NPAR	0.76	0.64–0.87	0.041^*^	22.5	67.7	70.8

PAB, Prealbumin; ALB, Albumin; WBC, White blood cell; NEU, Neutrophil; LAC, Lactate; NLR, Neutrophil-to-lymphocyte ratio; MLR, Monocyte-to-lymphocyte ratio; PLR, Platelet-to-lymphocyte ratio; NPAR, Neutrophil percentage-to-albumin ratio. Values marked with ^*^ *p* < 0.05, ^**^p < 0.01, and ^***^p < 0.001 indicate a statistically significant difference.

## Discussion

Inflammation-related markers vary depending on different inflammatory conditions. Rapid detection of inflammation and evaluation of prognosis in a patient is important when deciding on early treatment and can guide recovery (18). Therefore, various inflammation-related markers have been studied (12). In this study, we compared the usefulness of the plasma PAB concentration to that of other inflammation-related markers including the CRP concentration, albumin concentration, WBC count, NEU count, lactate level, NLR, MLR, PLR, and NPAR.

CRP is a positive APP that is synthesized in hepatocytes and is an excellent initial marker of inflammation (18). CRP has been considered one of the most “sensitive” markers of inflammation because it is useful to clinicians due to its involvement in inflammatory processes in both human and veterinary medicine (19–21). Recently, one study defined systemic inflammation in dogs with snake envenomation as a serum concentration of CRP > 35 mg/L (22). In another study, systemic inflammation at veterinary hospital admission was defined as a serum concentration of CRP > 35 mg/L within the first 24 h of presentation (13). In addition, several human medicine studies have shown that the plasma PAB concentration is significantly decreased in inflammation as defined by a cutoff CRP concentration (23, 24). Likewise, CRP has been used as a diagnostic criterion for demonstrate the degree of inflammation as well as sensitive marker of inflammation. Therefore, in this study, the plasma CRP concentration was used as a quantitative criterion of inflammation to evaluate the usefulness of PAB as an inflammatory biomarker.

The plasma PAB concentration was significantly lower in group B than in the control group and group A. PAB is known as a negative APP in human medicine (11). In inflammatory conditions, stimulation of cytokines such as interleukin-6 might reduce plasma PAB synthesis by increasing the synthesis of positive APPs such as CRP and serum amyloid A (15). In addition, the PAB concentration is decreased in inflammation due to transcapillary escape caused by increased vascular permeability (25). Likewise, a decreased PAB concentration in dogs with inflammation might be caused by a similar mechanism to that involved in human medicine (6–8, 15).

However, there were no significant differences in the PAB concentration in the control group and group A. A possible explanation for this result is that there was no significant inflammation in group A. Studies in human medicine have suggested that in patients without significant inflammation, inflammatory cytokines may not be significantly elevated enough to decrease the PAB concentration by reducing synthesis of PAB and increasing vascular permeability (16, 25). In this situation, PAB could be an alternative that reflects inflammatory conditions better than other inflammatory biomarkers in the presence of disease in dogs as well as humans.

In this study, the PAB and CRP concentrations showed the most significantly correlation among other inflammation-related parameters in dogs such as the albumin concentration, WBC count, NEU count, lactate level, NLR, MLR, PLR, and NPAR. In general, the CRP concentration is correlated with the severity of inflammation and a poor prognosis (9, 11). In addition, the plasma PAB concentration reflects the presence of persistent or resolving inflammatory states as effectively as CRP in human medicine (26). Therefore, these data suggest that the PAB concentration may reflect the severity of inflammation and thus have a potential use as a prognostic indicator in dogs.

Compared with the plasma albumin concentration, the plasma PAB concentration was significantly decreased in group B. Albumin is a negative APP synthesized in hepatocytes (18). The reasons for the deceased plasma albumin concentration in inflammation have been shown to be due to leakage from the intravascular compartment caused by increased vascular permeability, denaturation at sites of inflammation, reduced synthesis in the hepatic parenchyma, and degradation of albumin bound to toxins (12, 18, 27). Although albumin has been reported to be a useful, easy, and inexpensive parameter for detecting inflammatory diseases in dogs, it is a slow-reacting negative APP in dogs (18). On the other hand, PAB is used as a more sensitive inflammatory and prognostic marker than albumin because it has a shorter half-life than albumin in human medicine (16). Therefore, PAB may be used as a novel negative APP that can better reflect inflammation than albumin in veterinary medicine.

Lactate has been used as a parameter for evaluation of inflammation status in dogs (12, 28, 29). Hyperlactatemia is common in emergency and clinical critical care and occurs with evidence of tissue oxygen deficiency and systemic hypoperfusion (28). In human medicine, lactate has shown prognostic value in systemic inflammatory response syndrome (SIRS), and recent studies revealed that hypoxia, inflammation, viral infection, and tumors promote the production of lactate, which may act to modulate the immune-inflammatory response (29–31). In accordance with the findings of previous studies, lactate showed a significant difference between group B and the other groups (32, 33). In addition, there was significant difference between group A and healthy controls. Therefore, the plasma lactate concentration may reflect the severity of inflammation.

According to the ROC curve analysis, PAB showed a significant diagnostic capability for determining a cutoff value of 6.3 mg/L (89.5% sensitivity, 86.5% specificity) compared with the albumin concentration, WBC count, NEU count, lactate level, NLR, MLR, PLR, and NPAR. One possible explanation is that these hematological and biochemical parameters may have been affected by specific physiological effects. For instance, septic dogs could suffer from neutropenia and thrombocytopenia even in severe inflammatory conditions, which can reduce hematological and biochemical parameters such as the NLR and PLR (12). In contrast, PAB could have an advantage in identifying inflammation in various inflammatory conditions.

In this study, there were dogs with life-threatening diseases in group B such as acute pancreatitis, pyometra and malignant tumors with metastasis that might cause significant inflammation and death (10, 34–37). In contrast, group A included dogs with diseases with localized lesions that do not cause severe inflammation, such as mitral valve insufficiency, mammary gland tumors (WHO stage I) and intervertebral disk protrusion; these characteristics are similar to data previously reported in veterinary studies (18, 38, 39). According to our results, PAB was a good marker to differentiate group B from group A, and a decreased PAB concentration was present in dogs suffering from inflammation and was inversely correlated with the severity of inflammation. Therefore, it may be useful as an indicator in cases in which rapid therapeutic intervention must be assessed.

Recent human and veterinary medicine studies have revealed that evaluating both positive APPs and negative APPs in patients may be better for confirming the degree of inflammation and predicting survival (11, 27, 40, 41). For instance, during inflammation, the plasma CRP concentration increases, while the PAB concentration decreases. Consequently, the CRP-to-PAB ratio (CPR) merges the information of these two inflammation-related parameters into an index that is positively correlated with the degree of inflammation (16). The CPR is known to more accurately reflect a poor prognosis than the CRP or PAB concentration alone in human medicine (42–44). In this study, PAB was found to be a useful marker for inflammation as a negative APP in dogs. Therefore, both PAB and PAB-related inflammatory biomarkers (e.g., the CPR) could be useful inflammatory and prognostic biomarkers in veterinary medicine.

There are several limitations in this study. First, due to the limitation of the measurement method, a plasma PAB ≤ 3 mg/dL could not be measured. Therefore, CRP and PAB were not confirmed to be correlated even at a PAB ≤3 mg/dL. Second, the presence of anorexia and its duration in dogs in group B were not considered. Consequently, reduction in the PAB concentration due to malnutrition was not evaluated, which may have led to overestimated results in dogs with inflammation. In addition, since this study did not evaluate the survival rates of patients, a prognostic evaluation was not conducted.

In conclusion, this is the first study to demonstrate the clinical usefulness of the plasma PAB concentration as an inflammatory marker in dogs. The present study showed that PAB is a useful inflammatory marker in dogs. Therefore, measuring the plasma PAB concentration along with the CRP concentration may be more useful for evaluating inflammation than measuring the CRP concentration alone in patients. Use of this marker could be one option for enhancing the diagnostic accuracy of detecting inflammation by veterinarians in clinical fields. Further studies are needed to examine the variation in the plasma PAB concentration during clinical courses and to assess its relationship with disease severity to determine its potential as an inflammatory marker.

## Data availability statement

The original contributions presented in the study are included in the article/supplementary material, further inquiries can be directed to the corresponding author.

## Ethics statement

This prospective investigation was approved by Chonnam National University Institutional Animal Care and Use Committees (IACUC) and undertaken under written informed client consent (Approval number: CNU IACUC-YB-2022-110). Written informed consent was obtained from the owners for the participation of their animals in this study.

## Author contributions

S-WP and KK were involved in the study design, sample collection, and laboratory analysis and were responsible for writing the manuscript. O-KK was involved in the draft preparation and laboratory analysis. W-BR and C-ML were involved in the study design and revision of the manuscript. All authors contributed to the article and approved the submitted version.

## Funding

This work was supported by the National Research Foundation of Korea (NRF) grant funded by the Korean government (MSIT) (no. 2022R1F1A1059721) and Korea Institute of Planning and Evaluation for Technology in Food, Agriculture and Forestry (IPET) and Korea Smart Farm R&D Foundation (KosFarm) through the Smart Farm Innovation Technology Development Program, funded by Ministry of Agriculture, Food and Rural Affairs (MAFRA) and Ministry of Science and ICT (MSIT), Rural Development Administration (RDA) (no. 421015–04).

## Conflict of interest

The authors declare that the research was conducted in the absence of any commercial or financial relationships that could be construed as a potential conflict of interest.

## Publisher’s note

All claims expressed in this article are solely those of the authors and do not necessarily represent those of their affiliated organizations, or those of the publisher, the editors and the reviewers. Any product that may be evaluated in this article, or claim that may be made by its manufacturer, is not guaranteed or endorsed by the publisher.

## Abbreviations

APP: Acute phase protein
AUC: Area under curve
CBC: Complete blood count
CRP: C-reactive protein
MLR: Monocyte-to-lymphocyte ratio
NEU: Neutrophil
NLR: Neutrophil-to-lymphocyte ratio
NPAR: Neutrophil percentage-to-albumin ratio
PAB: Prealbumin
PLR: Platelet-to-lymphocyte ratio
ROC: Receiver operating characteristic
WBC: White blood cell
